# Regionally accentuated reversible brain grey matter reduction in ultra marathon runners detected by voxel-based morphometry

**DOI:** 10.1186/2052-1847-6-4

**Published:** 2014-01-17

**Authors:** Wolfgang Freund, Sonja Faust, Christian Gaser, Georg Grön, Frank Birklein, Arthur P Wunderlich, Marguerite Müller, Christian Billich, Uwe H Schütz

**Affiliations:** 1 Department of Diagnostic and Interventional Radiology, University Hospital Ulm, Albert-Einstein-Allee 23, 89081 Ulm, Germany; 2 Departments of Psychiatry and Neurology, Jena University Hospital, Jahnstraße 3, 07743 Jena, Germany; 3 Section Neuropsychology and Functional Imaging, University Hospital Ulm, Leimgrubenweg 12-14, 89073 Ulm, Germany; 4 Department of Neurology, Mainz University Medical Center, Langenbeckstraße 1/503, 55131 Mainz, Germany; 5 Outpatient Rehabilitation Center at University Hospital Ulm, Pfarrer-Weiß-Weg 10, 89077 Ulm, Germany

**Keywords:** Voxel based morphometry, VBM, Catabolism, Plasticity, Brain, Default mode network, MRI, Ultra marathon

## Abstract

**Background:**

During the 4,487 km ultra marathon TransEurope-FootRace 2009 (TEFR09), runners showed catabolism with considerable reduction of body weight as well as reversible brain volume reduction. We hypothesized that ultra marathon athletes might have developed changes to grey matter (GM) brain morphology due to the burden of extreme physical training. Using voxel-based morphometry (VBM) we undertook a cross sectional study and two longitudinal studies.

**Methods:**

Prior to the start of the race 13 runners volunteered to participate in this study of planned brain scans before, twice during, and 8 months after the race. A group of matched controls was recruited for comparison. Twelve runners were able to participate in the scan before the start of the race and were taken into account for comparison with control persons. Because of drop-outs during the race, VBM could be performed in 10 runners covering the first 3 time points, and in 7 runners who also had the follow-up scan after 8 months. Volumetric 3D datasets were acquired using an MPRAGE sequence. A level of p < 0.05, family-wise corrected for multiple comparisons was the a priori set statistical threshold to infer significant effects from VBM.

**Results:**

Baseline comparison of TEFR09 participants and controls revealed no significant differences regarding GM brain volume. During the race however, VBM revealed GM volume decreases in regionally distributed brain regions. These included the bilateral posterior temporal and occipitoparietal cortices as well as the anterior cingulate and caudate nucleus. After eight months, GM normalized.

**Conclusion:**

Contrary to our hypothesis, we did not observe significant differences between TEFR09 athletes and controls at baseline. If this missing difference is not due to small sample size, extreme physical training obviously does not chronically alter GM.

However, during the race GM volume decreased in brain regions normally associated with visuospatial and language tasks. The reduction of the energy intensive default mode network as a means to conserve energy during catabolism is discussed. The changes were reversible after 8 months.

Despite substantial changes to brain composition during the catabolic stress of an ultra marathon, the observed differences seem to be reversible and adaptive.

## Background

In 2009 (April 19th to June 21st) the TransEurope-FootRace 2009 (TEFR09) took place. It was the second European transcontinental multistage ultra marathon race and covered 4487 km (2788 miles) from Bari in the south of Italy to the North Cape. A group of 68 endurance runners with a mean age of 50.5 years ranging from 26 to 74 and encompassing 11 women and 57 men from 12 nations met the challenge. Their goal was to run the distance in 64 days without a day rest. Thus, they expected to complete an average distance of 70.1 km daily, that is 1.7 marathon distances per day (minimum: 44 km/d, maximum: 95.1 km/d) for 64 consecutive days without any day rest [1]. The race and the research project [2] as well as some results have been described elsewhere [3-5].

Brain anatomy is not static and changes due to structural plasticity have been described in several previous studies [6,7]. For example, hand inactivity related volume loss of the motorcortex has been observed to be reversible after reuse of the hand [8]. Sport related grey matter (GM) volume increase has been demonstrated in premotor and parietal regions of golfers [9] and has also been reported to correlate with aerobic capacity [10].

Contrary to the described activities, walking and running is challenging only during the period of learning it. The process of running is highly automated after childhood [11] without relevant challenges for motor coordination or intellectual capabilities. Functional magnetic resonance imaging (fMRI) studies have shown that the control of running is associated with activity in spinal motor generators under supraspinal (e.g. cerebellar) control [12,13] but with only marginal cortical involvement. Thus, gross plastic structural changes of brain regions relevant to the motor aspects of running could not be expected from participation in an ultra marathon.

Rather we speculated that participation in the TEFR09 would prove challenging for other brain functions such as pain suppression with possible activation of amygdala – hippocampal or perigenicular cingulate areas [14-16] or for sheer will power with possible activation of prefrontal areas [17].

To observe cerebral effects of sustained metabolic and functional challenges in TEFR09 participants, we had planned a voxel based morphometry (VBM) [18] study to investigate voxel-wise differences of brain anatomy between TEFR09 participants and controls at baseline, and also a longitudinal study to observe changes in brain anatomy due to the participation in the TEFR09.

## Methods

### Subjects

After approval of the local ethics committee (University of Ulm, decision 78/08-UBB/se) and in accordance with the Declaration of Helsinki, 13 athletes without any contraindications to MRI were recruited signing informed consent. We had planned to perform brain scans before, twice during, and 8 months after the race. Due to the strong challenge and ensuing phenomena of fatigue, compliance of runners to participate in MR imaging in the evening after a day of running was limited and resulted in drop-outs such that data of a reduced number of runners could be included into the analysis of the longitudinal data. We carried out a cross sectional comparison of athletes and controls with 12 subjects (one of the 13 participating athletes was not able to participate in the baseline scan). We also performed a longitudinal VBM analysis with ten runners covering 3 time points and a second analysis with seven runners who were able to attend a follow-up scan after 8 months at time point 4. All included TEFR09 participants were men, aged 31 to 67 years with a mean of 50.2 (SD 9.7) years. In the year before the race, they had trained 12.9 hours/week (SD 3.4).

### Controls

We individually matched 12 men aged 30 to 70 years with mean age of 49.5 (SD 10.6) years into the control group. Inclusion criteria for the control group were matched sex, handedness and age (± 3 years). Exclusion criteria were a chronic pain disorder (neurological interview) and any participation in long distance runs (e.g. marathon runs) during the previous five years. The controls had performed sports for 2.5 hours/week (SD 1.4).

### MR acquisition protocol

Scanning was performed on three identical 1.5 T Siemens (Erlangen, Germany) MAGNETOM Avanto® MR scanners with identical sequence parameters. Two scanners were located at our university. The third was a mobile MRI unit mounted on a truck escorting the runners during the race. Volumetric 3D datasets were acquired using an MPRAGE sequence with the following sequence parameters: Time of repetition/echo/inversion TR/TE/TI was 2100/4.8/1060 ms, flip angle FA 15° and isotropic voxel-size of 1 mm. Direction of acquisition was sagittal with 192 slices covering the entire brain of each participant.

### Cross sectional design

Scanning was performed on one scanner for all participants except one TEFR09 participant who was scanned on the mobile unit. The TEFR09 participants were scanned before the race, the controls within three months after starting the race.

### Longitudinal design

The first examination (time point 1, t1) took place before the start of the race (same data as used in the cross sectional design). Time points 2 and 3 (t2 and t3) were planned at 2400 and 4000 km and the follow-up was performed on average 8 months after the end of the race.

Scanning data from 10 runners were available for the analysis comparing time points t3-t1, t2-t1 and t3-t2. Seven runners were available for time point 4 (t4) follow-up scan and were contrasted to time points t1, t2 and t3, respectively.

In a previous analysis, we had shown that the sample of 10 subjects attending 3 examinations and the sample of 7 subjects attending all 4 examinations did not differ with respect to biometrical data, and both groups were representative of the whole group of TEFR09 participants [3]. Furthermore, we had shown that the physical load of the multi stage ultra marathon had resulted in profound changes of body weight and total brain volume [3] and that both samples of athletes exhibited similar changes.

### Data analysis

Data preprocessing and analysis was performed with the VBM8 toolbox (http://dbm.neuro.uni-jena.de/vbm), which is incorporated in the SPM8 software (http://www.fil.ion.ucl.ac.uk/spm/) running on MATLAB® (Mathworks). Within the same generative model, images were corrected for bias-field inhomogeneities, registered using linear (12-parameter affine) and nonlinear transformations using DARTEL (Diffeomorphic Anatomical Registration Through Exponentiated Lie Algebra) [19], and tissue-classified into GM, white matter (WM), and CSF. This “new segmentation” procedure was further refined by accounting for partial volume effects [20], by applying adaptive maximum a posteriori estimations [21], and by applying a hidden Markov random field model [22] as described by Gaser et al. [23].

### Cross sectional design

Data were preprocessed using the standard batch for cross-sectional data as described above (including modulation). Finally, segmentations were smoothed using a 10 mm full-width at half maximum (FWHM) Gaussian kernel. A two-sample *t*-test was performed to test for local volume differences between athletes and controls in both directions. Statistical threshold was set to p < 0.05, and corrected for multiple comparisons using family wise error (FWE). Only cluster exceeding a size of more than 15 voxels are reported. To avoid possible edge effects between different tissue types, all voxels with GM values of < 0.2 were excluded (absolute threshold masking).

### Longitudinal design

The preprocessing of the imaging data before the statistical analysis was carried out using a specific batch for longitudinal data as implemented in VBM8 (there was no modulation used because it is not necessary in longitudinal designs where the focus is on relative differences between the same objects). Individual T1w images were first aligned to a T1 template in MNI-space (Montreal Neurological Institute) in order to bring them in a common reference frame with respect to translation and rotation. A mean image was calculated from these realigned images and a first realignment of raw data followed enclosing this mean image as a reference. At this stage individual images were bias-corrected to account for signal inhomogeneities. The resulting mean image was segmented into grey matter (GM), white matter (WM) and cerebrospinal fluid (CSF) using the segmentation approach described above. The segmented GM was normalized using DARTEL (to the same template as in the cross sectional design). The resulting normalization parameters were applied to the realigned and bias-corrected single images. Afterwards, these images were segmented and a second realignment followed. Smoothing of GM segments was done using a 10 mm FWHM Gaussian kernel (suitable for small samples sizes [24]) and data were passed to the statistical analysis.

We used an ANOVA with a flexible factorial design with factors ‘subject’ and ‘time’. In the first longitudinal study with the 10 runners group, one model was set up with 10 runners and GM segments at 3 time points. The factorial design for 10 runners was masked as described below to exclude scanner effects, so no covariate was needed. For the second longitudinal study, we used the 7 runners group with 4 time points including the follow up. The model for 7 runners considered the fact that data were obtained from three different scanners using a covariate coding for each MR scanner.

Again, an absolute threshold masking with a threshold of 0.2 was applied to avoid possible edge effects between different tissue types. To infer a significant decrease over time in the factorial design comprising three time points a t-contrast was formulated comparing GM segments at time point 3 against those of baseline (time point 1 minus time point 3). Statistical threshold for this contrast was set to level of p < 0.05, family-wise corrected to account for multiple comparisons.

To ascertain a true linear trend and to exclude possible systematic errors due to different scanner types, this contrast was further inclusively masked by the conjunction of two additional t-contrasts inferring significant differences between time points 2 and 1, and between time points 3 and 2. Voxels were included only if the conjunction of both contrasts passed a significance level of p < 0.001 (uncorrected) to account for the one-sidedness of these t-contrasts. The inverted main contrast (time point 3 minus time point 1) was assessed for the sake of completeness (no significant results). The factorial design including 7 runners was mainly used to contrast time points 1–4 and 4–1 in order to test whether there were significant changes in GM segments before and 8 months after the end of TEFR 2009, but the contrasts of the other time points are also reported.

The resulting coordinates of significant voxels were transformed from MNI space into Talairach space using GingerAle 2.1 incorporating the Lancaster transformation [25]. The anatomical analysis was conducted with Talairach Client Version 2.4.2 [26]. Significant effects were projected on a mean image of all 10 runners from time point 1. 3D rendering was performed with MRIcron Version April 2010 [27].

## Results

### Cross sectional comparison

There were no significant grey matter volume differences between runners in preparation for an endurance run and controls.

### Longitudinal study

At time point 2 the runners had finished 2475 km on average, and 4001 km at time point 3. The average time elapsed between the end of the race and the follow-up measurement was 256 days.

#### Regional VBM GM volume changes

**Figure 1:**
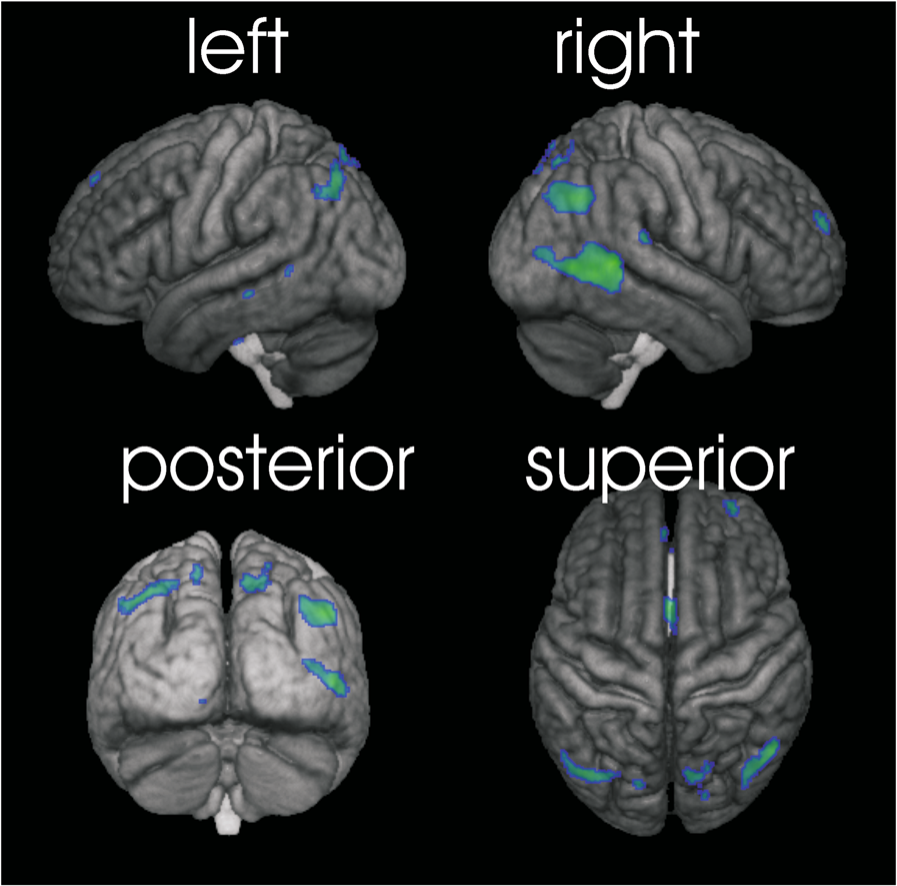
3D rendering of the surface projection of clusters of significant volume decrease from start to time point 3 at a mean of 4001 km (contrast t3-t1 with masking as described, p < 0.05 FWE corrected).

**Figure 2:**
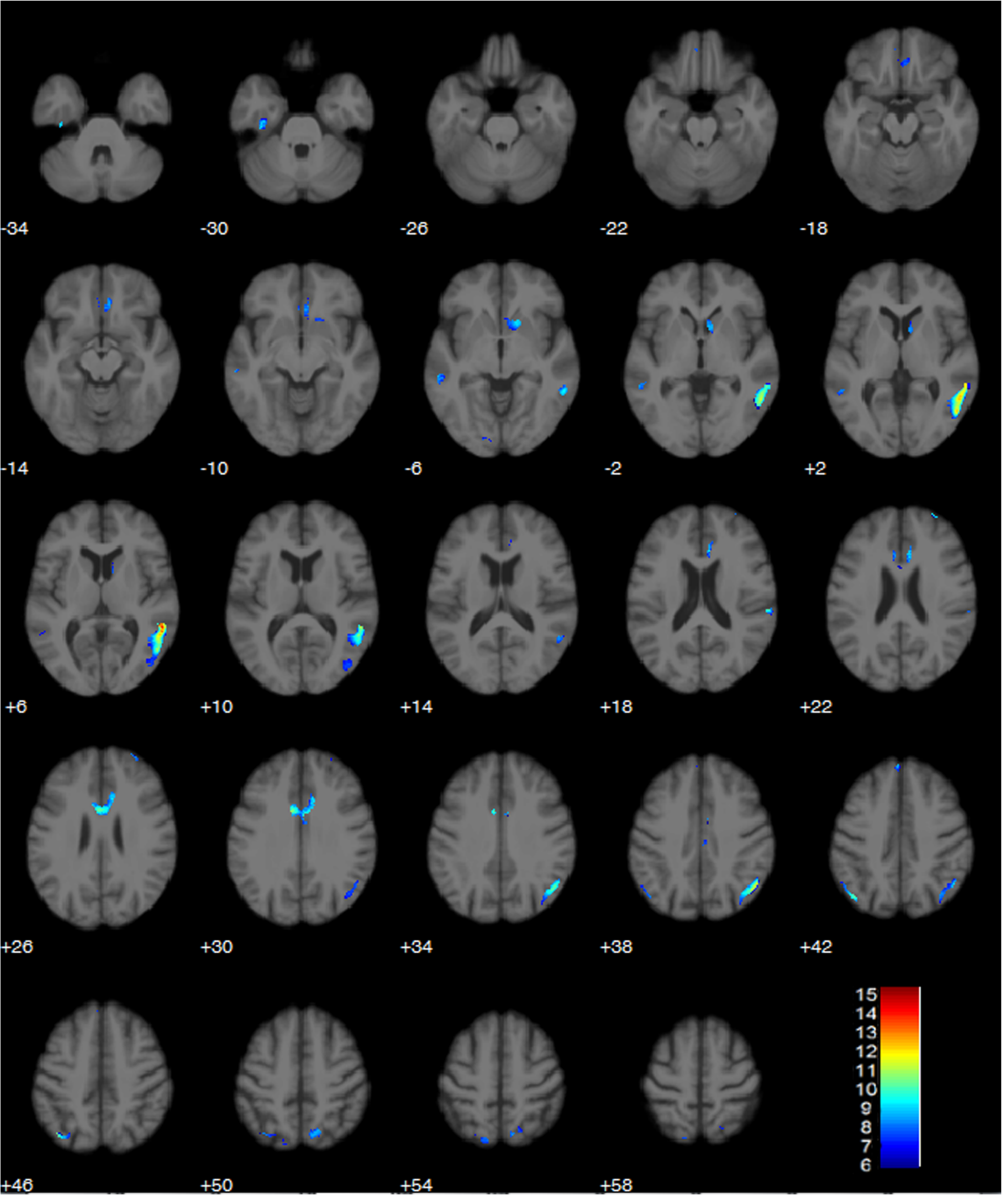
Axial slices showing clusters of significant volume decrease from start to time point 3 at a mean of 4001 km (contrast t3-t1 with masking as described, p < 0.05 FWE corrected).

The ANOVA with the flexible factorial design comprising three time points in 10 runners revealed a highly significant effect of time, and showed a strong linear decrease of regional GM volumes in anatomical locations summarized in Table 1 (p < 0.05 FWE). Due to the inclusive masking procedure outlined in the Methods section this linearly decreasing trend was further characterized by significant (p < 0.001) decreases between consecutive time points (t2-t1 and t3-t2). Significant clusters were located mainly bilaterally in the posterior temporal and occipitoparietal cortices as well as in anterior cingulate cortex and right ventral caudate nucleus (see Table 1 and Figures 1 and 2).

**Table 1:** Clusters of significant volume changes in contrast t3-t1, describing GM volume decrease from start to time point 3 at a mean of 4001 km

**Functional region**	**Anatomical localisation**	**Side**	**Brodmann area**	**x**	**y**	**z**	**z-score**
Bilateral temporal cortex and parietal lobe	Medial temporal gyrus	Right	21	51	-43	7	6.60
			37	45	-62	2	6.35
			22	54	-39	1	6.35
	Superior temporal gyrus and postcentral gyrus, parietal lobe	Right	42, 40	56	-30	19	5.73
	Medial temporal gyrus	Left	21	-56	-29	-7	5.59
			21	-53	-37	-5	5.39
			21	-48	-42	0	5.39
	Uncus	Left	20	-34	-19	-28	5.69
Bilateral occipitoparietal cortex	Angular gyrus	Right	39	46	-51	33	6.07
	Precuneus	Right	19	32	-68	34	5.47
	Precuneus	Right	7	14	-68	41	5.51
			7	8	-72	45	5.51
			19	15	-81	38	5.42
	Precuneus		19	-40	-71	38	5.96
	Angular gyrus	Left	39	-48	-62	34	5.29
	Precuneus		7	-24	-73	43	5.08
	Precuneus	Left	7	-15	-75	48	5.20
	Lingual gyrus	Left	18	-15	-85	-9	5.12
Bilateral ACC/perigenual cingulate cortex adjacent medial prefrontal cortex	Cingulate gyrus	Left	24	-3	4	26	6.15
		Left	24	-7	1	34	5.96
		Right	24	1	-3	31	5.68
	Cingulate gyrus	Right	31	1	-27	35	5.16
	Superior frontal gyrus	Left	8	-3	39	46	5.43
	Superior frontal gyrus	Right	9	27	51	29	5.81
	Medial frontal gyrus	Left	10, 11	-3	44	-11	5.13
Right caudate head	Caudate		Caudate head	13	12	0	5.50
	Caudate	Right	Caudate head	6	7	3	5.42
	Anterior cingulate		24	5	27	-4	5.36

Masking as described in the method section, p < 0.05 FWE corrected. In the columns x, y and z brain coordinates in stereotactic MNI-space are listed. Z-score refers to the standard norm distribution’s z-statistic.

The ANOVA computed with the second factorial design of the 7 runners investigated at four time points (now including the follow-up) showed the same linearly decreasing effect (p < 0.001 for masking with t2-t1 and t3-t2 and p < 0.05 FWE for t3-t1) from t1 to t3 as observed in the investigation of the ten runners at 3 time points. Comparing regional GM volumes obtained at t1 with those obtained at follow-up did not show any significant differences. Interestingly, all regions that had demonstrated a significant decrease from t1 to t3 showed a significant increase from t3 to t4 at a level of p < 0.05 FWE (no masking).

## Discussion

The main findings of our study were GM volume decreases mainly located in bilateral posterior temporal and occipitoparietal brain regions, in the anterior cingulate cortex and in the right ventral caudate nucleus during participation in an ultra marathon. These changes were accompanied by decreasing body weight and global GM matter reduction during the race [3]. All changes reversed to baseline when measured eight months after finishing the race.

In a cross sectional comparison we found no significant differences of regional grey matter distribution between our rather moderately physically active controls and the TEFR09 participants. This is at first surprising given the fact that both groups practise sports differently [4] and could be associated with low sensitivity due to our small sample sizes [24]. Still, repetitive running might not activate specific brain areas enough - in contrast to e.g. juggling [6] - to induce local structural plasticity despite tremendous differences in training load. Therefore we could not elucidate any predispositions for or consequences of ultra marathon training regarding brain plasticity. In a previous study [3] we have already shown that substantial structural brain lesions did not occur during the TEFR09 participation. Together with the present results one might therefore conclude that ultra marathon training does not permanently alter brain anatomy.

However, our previous longitudinal analyses have already shown that a reversible global volume loss of brain grey matter occurred during the TEFR09 [3]. We are now able to add that this global GM volume loss is not evenly distributed but has a specific spatial distribution. As a putative mechanism of action for global GM loss catabolism with likely loss of proteins has been described which is further supported by the apparently strong parallel variation of body weight and GM volume which both returned to baseline after eight months in our previous study [3]. Other reports on multi stage endurance events also showed catabolism [28,29].

In the present longitudinal analyses focussing on localized GM volume changes we found four different brain regions with significant GM reduction: the bilateral posterior temporoparietal cortex, bilateral occipitotemporal cortex, bilateral anterior cingulate cortex and adjacent prefrontal cortex, and the head of the right caudate nucleus.

Recently, a series of studies have reported changes of GM that were associated with behavioural changes. For example, regional changes of GM indicating structural plasticity with reversible (upon treatment) GM decrease have been shown with VBM for pathologic states such as immobilization [8], pain syndromes [30], obsessive compulsive disease [31], but also action induced GM increase for sports like juggling [7] and playing golf [9]. However, these studies differ from the present one since the behaviour under investigation could be experimentally varied so that variations of behaviour could be related to structural brain changes. This was not the case with the present naturalistic study. Therefore any attempt to give an explanation for the present distribution of regional GM changes is speculative and great caution in discussion is necessary [32]. Nevertheless, some of the authors of the present study accompanied the race and were therefore in the position to experience the uniformity and monotony of daily running such long distances. Thus, some of the decreases of GM volume in different brain areas can be discussed under the assumption of mental underload. For example, the bilateral occipitotemporal cortex (encompassing Brodmann’s Area (BA) 18, 19, 39 and 7) are well known to serve visual and visuospatial processing [33-35]. Therefore, one might assume that daily running on streets in straight lines may impose only little needs on detailed visual processing. This assumption might be supported by vertigo imaging studies, which have repetitively shown that the parietooccipital cortex is deactivated when visual acuity is not necessary [36]. Similarly, GM volume reductions in bilateral posterior temporal cortex and parietal lobe (BA 20–22, 37, 40 and 42), which include auditory association areas [33,37,38], might be related to running the entire day in isolation with reduced auditory stimulation. The anterior cingulate gyrus (BA 24, 31) together with prefrontal cortices (BA 8–11) is thought to play a crucial role in attentional and inhibitory control [39] and more generally in cognitively demanding tasks [40]. All these skills are less demanded during daily running and therefore these areas may have received less priority in maintenance under conditions of severe catabolism [41]. The caudate nucleus has been implicated in motor planning, especially in demanding conditions [42] which were also absent during the run.

Yet another interpretation of regional GM volume reductions can be derived when considering the similarity between their anatomical distribution and those brain areas that belong to the so called default mode network (DMN). Essentially, the DMN comprises the temporoparietal junction, the inferior parietal lobule, posterior cingulate cortex and lateral temporal cortices. This network of brain areas is thought to be associated with self oriented thinking, and is deactivated during externally oriented attention [43,44]. It has been calculated that roughly 60-80% of the brain’s high energy consumption is used in baseline activity [45], possibly permitting faster responses in cases of functional challenges than a truly resting brain without activity [46]. During ultra marathon running most of the day a resting state system might be less important so that a reduction of GM volume could result. However, an important area of the DMN, the posterior cingulate cortex PCC [47] was not involved in GM reduction, and the caudate nucleus (showing volume decrease in our data) has not been reported as a member of the DMN so far.

### Limiting factors and technical considerations

One of the major limiting factors of the present study is small sample size, which could condition false negative results (mainly the negative results in the cross sectional study) [24]. Although a larger sample of athletes willing to participate in our examinations could be recruited at the beginning of the race, the rather time consuming examinations as an add-on to the already tight time schedule for athletes performing at their limits has prohibited inclusion of more subjects.

We did not perform modulation in the preprocessing of our VBM data in the longitudinal studies because it has recently been shown that unmodulated images improve sensitivity [48] to detect mesoscopic changes, such as cortical thinning, which are probably those expected in longitudinal VBM comparisons. However, in the cross sectional study modulation was performed.

Since the MRI scanner mounted on the truck was available only for the run, scanning before the race and during follow-up had to be performed on different scanners. A third scanner became necessary due to the long follow-up interval. MRI scanners were identical models and used identical sequence parameters. Usually, great care has to be taken to avoid systematic errors in longitudinal VBM comparisons, so that data acquisition should not be performed on different scanners. However, we could already show that the use of different identical scanners did not affect the global volume changes in our setting [3]. For the present investigation with regional volume changes we have tried to protect the longitudinal analyses against putative influences from the use of different scanner types by the inclusive masking procedure designed to accept only changes verified on same scanner comparisons. By this masking operation, inference of significant volume changes from t1 to t3 was constrained by significant changes between t2 and t3 for which the data had been scanned on the same truck mounted scanner only. Since the relevant contrast of t3 against t1 could only infer significant results for those voxels significant in the t3 to t2 comparison (same scanner), present results were deemed to not having been influenced by a systematic bias stemming from different scanner types.

## Conclusion

The accentuated and regionally distributed GM volume reduction in our study may be associated with decreased demands during daily running of very long distances in areas responsible for higher brain functions. Furthermore, the need for energy conservation may drive the reduction of high energy consuming default mode network circuits. As possible mechanism of action catabolic processes appear as candidates who should be addressed in future investigations. Extreme running may serve as model to better understand mechanisms involved in transient regional brain volume reductions. It may show mechanisms of the brain to cope with extreme catabolic demands encountered also in cancer and its treatment. The reversibility of the changes at follow-up supports the finding that GM changes in chronic diseases like chronic pain are not related to structural atrophy or neuronal loss but are functional and reversible [30]. Accordingly relevant and persisting long term effects on the brain’s integrity in ultra-marathon runners are less likely to occur. Still, this conclusion awaits further research and replications.

## Abbreviations

DARTEL: Diffeomorphic anatomical registration through exponentiated lie algebra; GM: Gray matter; MRI: Magnetic resonance imaging; TEFR09: TransEurope-footrace 2009; t1: Time point 1; t2: Time point 2 et cetera; t1-t2: A contrast involving the comparison of t1 and t2, showing regions significantly larger at t1 than at t2; VBM: Voxel based morphometry; WM: White matter.

## Competing interests

The authors declare that they have no competing interests.

## Authors’ contributions

WF proposed and designed the study, worked on the statistical analysis, interpreted the results and wrote the report. SF planned the statistical analysis of the volumetric data and performed it, and wrote parts of the report. CG revised the methodology of the statistical analysis and wrote parts of the paper. GG contributed to the statistical analyses of the brain and non-brain data and wrote parts of the report. FB helped to conceive and plan the study and wrote parts of the paper. APW planned the study and the physical properties of the used sequences and wrote part of the manuscript. MM took part in the statistical analysis and revised the paper. CB planned the study and sampled data. UHS planned the TEFR09 project and this study and sampled data and read the images. All authors critically revised and approved the final manuscript.

## Pre-publication history

The pre-publication history for this paper can be accessed here:

http://www.biomedcentral.com/2052-1847/6/4/prepub
